# Again in Harness

**Published:** 1891-12

**Authors:** 


### Again in the Harness.

Most of the dental profession of this country know, either directly or indirectly, "*The Dental Advertiser*," of Buffalo, N. Y. This has been a quarterly journal, published for many years by the Buffalo Dental Manufacturing Company, and was edited by one of the proprietors of that establishment. A change in its editorial management was inaugurated with the October number, which included making Dr. W. C. Barrett, M.D., D.D.S., the editor in charge; and we presume, pretty much everybody will understand what that means. As is well known, Dr. Barrett, was the proprietor and editor of the "*Independent Practitioner*" for a number of years, and it is well known how lively he made that journal, and with what energy he stirred up things generally and some few things in particular. We will here suggest, that if any have corns, it would be well to keep out of his way, for he has a broad foot, and is by no means a light weight, and when that member comes down it means something. The Doctor is a fearless and independent writer, and we are glad to welcome him again into the editorial ranks, fully realizing that he is a *large* addition to it.

